# Distribution of *Benthesicymus tanneri* Faxon, 1893 (Dendrobranchiata, Benthesicymidae) off the west coast of Mexico and notes on its morphology

**DOI:** 10.3897/zookeys.473.8956

**Published:** 2015-01-20

**Authors:** Michel E. Hendrickx, Vanesa Papiol

**Affiliations:** 1 Laboratorio de Invertebrados Bentónicos, Unidad Académica Mazatlán, Instituto de Ciencias del Mar y Limnología, Universidad Nacional Autónoma de México, P.O. Box 811, Mazatlán, Sinaloa, 82000, Mexico

**Keywords:** *Benthesicymus tanneri*, eastern Pacific, distribution, key to species

## Abstract

A large series of specimens of *Benthesicymus tanneri* Faxon, 1893 (Dendrobranchiata; Benthesicymidae) was collected during an extensive survey of deep-water invertebrate fauna off western Mexico. In total, 61 males and 122 females (M:F ratio = 1:2) from 44 sampling stations were examined, considerably increasing the number of known specimens and sampling localities for this species which is widely distributed along the Pacific coast of Mexico. The collection is the largest available for this species to date and presents first records from off the west coast of the Baja California Peninsula and a slight increase of the northernmost record within the Gulf of California. On the whole, females grew larger than males. The petasma of males of different sizes and the female thelycum of *Benthesicymus tanneri* are illustrated. The petasma of *Benthesicymus tanneri* presents a ventrolateral crescent-shape process otherwide found only in *Benthesicymus tirmiziae* Crosnier, 1978 and in *Benthesicymus bartletti* S.I. Smith, 1882. A key to the four species of *Benthesicymus* presently known from the eastern Pacific is presented.

## Introduction

Benthesicymidae is a relatively large family of shrimps that contains 39 species within five genera. The most species-rich genera are *Gennadas* (16 species) and *Benthesicymus* (15 species) (De Grave and Fransen 2011). The genus *Benthesicymus* was first reviewed by Burkenroad (1936) when the group comprised 19 species. However, since then several of those species have been considered junior synonyms or assigned to different genera, and three new species have been described (see De Grave and Fransen 2011). Burkenroad (1936) separated the 19 species known at that time into two groups (Groups I and II) taking into consideration a long series of characters, including the shape of the exopod of the first maxilliped, the merus of the second maxilliped and the dactyl of the third maxilliped, the relative size of the exopodite of the pereiopods, the position of the pterygostomial spine, and the shape of the pterygostomial carina. In addition, he also considered the structure of the petasma and thelycum. According to Burkenroad (1936), the type species of *Benthesicymus*, *Benthesicymus crenatus* Spence Bate, 1881, is part of Group I. The two groups (I and II) are essentially the same as those referred to by Kikuchi and Nemoto (1991) and Dall (2001), but these authors used a reduced series of characters and an updated list of species. Kikuchi and Nemoto (1991), however, ommitted *Benthesicymus cereus* Burkenroad, 1936, from their list and key, and included *Benthesicymus longipes* Bouvier, 1906 (now synonymized with *Benthesicymus iridescens* Spence Bate, 1881) and *Benthesicymus brevirostris* Kikuchi & Nemoto, 1991 (now transferred to the genus *Altelatipes*). Dall (2001) cited the 15 species from the Indo-West Pacific known to him, including *Benthesicymus brevirostris* and *Benthesicymus longipes*, and provided a key to species from that region.

Characters used by Kikuchi and Nemoto (1991) in their definition of Group I and II included the position of the branchiostegal spine, the shape of the second maxilliped and of the dactylus of third maxilliped, and the relative size of pereiopods’ exopod. Their Group II includes five species, two of which have been recorded in deep waters of the Mexican Pacific: *Benthesicymus altus* Spence Bate, 1881, and *Benthesicymus tanneri* Faxon, 1893 (see Hendrickx 1996). Although similar in their general shape, *Benthesicymus altus* and *Benthesicymus tanneri* are easy to separate based on the structure of the thelycum and petasma. Kikuchi and Nemoto’s (1991) Group I included 10 species, one of them also reported off western Mexico, *Benthesicymus laciniatus* Rathbun, 1906, which distinctively features small spines on the posterolateral margin of the fifth abdominal somite.

To date, four species have been certainly recorded in the eastern Pacific. *Benthesicymus altus* is distributed from California, USA, to the Galapagos Islands, but it also occurs in the Atlantic and Indo-Pacific (Guzmán and Wicksten 2000). *Benthesicymus tanneri* is known from California, USA, and the Gulf of California (north to 27°34'N; 110°53'W), Mexico, to Chile (21°19'S) (Retamal and Soto 1993; Wicksten and Hendrickx 2003). The taxonomic status of *Benthesicymus laciniatus* Rathbun, 1906, was reviewed by Wicksten (2004) and this species is known from Hawaii, Santa Catalina Island (as *Gennadas pectinatus* Schmitt, 1921, a junior synonym of *Benthesicymus laciniatus*), California, USA, and off Baja California Peninsula (31°20'N; 120°8'W) (Wicksten 2004). Another species of Group II, *Benthesicymus investigatoris* Alcock & Anderson, 1899, is widely distributed in the world oceans and has been reported in the eastern Pacific off Chile (Salas y Gómez Island and Ridge; Nazca Ridge) by Retamal and Moyano (2010). There is an additional record for a fifth species of *Benthesicymus* in the eastern Pacific, *Benthesicymus crenatus*, but this record is based on a tentative identification by I. Peréz-Farfante (“Benthesicymus cf. crenulatus”, USNM 216490) from a specimen collected next to the Cortés Bank (32°08'N; 120°48'W; 3782 m depth) and it is doubtful considering that all records for *Benthesicymus crenatus* are in the northwestern and central Pacific Ocean (Jamieson et al. 2009).

*Benthesicymus tanneri* is a moderate large species, with females reaching up to 99 mm total length (Hendrickx 1996) and a maximum known size of 112 mm (Faxon 1893). Material examined by Faxon (1893) was collected in 22 “Albatross” stations, from off Ecuador (3°56'N; 81°40'15"W) to the Central Gulf of California (27°34'N; 110°53'40"W), in a depth range from 385 to 1322 fathoms (ca 704–2,427 m depth). Because it is a deep-water species, records after those presented by Faxon (1893, 1895) are scarce and several authors have only repeated previous literature records or geographic distribution (e.g., Schmitt 1921, Rodríguez de la Cruz 1987, Wicksten 1989, Wicksten and Hendrickx 1992, 2003, Hendrickx 1993, 1995, Guzmán and Wicksten 2000). Rathbun (1904: 147) was the first to report on additional material collected by the “Albatross” off San Diego, within the Gulf of California, and off Ecuador, including the Galapagos Islands (Sts. 2923, 2929, 3009, 3010, 2792, 2793, 2808, 2818; from 331 to 1322 fathoms). In her monograph on shrimp from Peru, Méndez (1981: 31) included a large series of samples collected from a very wide latitudinal range (i.e., 3°31'S to 18°17'S) between 500 and 1300 m depth. Kameya et al. (1997) reported *Benthesicymus tanneri* in three stations off Peru, Retamal and Jara (2002) cited it from off Chile, and Cornejo-Antepara (2010) from off Ecuador. It is also known from off Costa Rica (Vargas and Wehrtmann 2009) and off El Salvador (J. López, pers. comm.).

Material collected in Mexican waters during the TALUD cruises III-VII (1991–2001) in the SE Gulf of California was reported by Hendrickx (2001) and Hendrickx (2004; distribution maps), adding many new records and increasing the known distribution range of this species. A large series of specimens, however, was collected during subsequent research cruises off the Pacific coast of Mexico and has not yet been reported. This series is included herein. This contribution provides and updated distribution of *Benthesicymus tanneri* for the Mexican Pacific and new data related to the petasma and thelycum of this species. Additionally, a taxonomic key for the species occurring in the American Pacific is provided.

## Material and methods

The material on which this study is based was collected by the R/V “El Puma” of the Universidad Nacional Autónoma de México (UNAM), between 1991 and 2014. Specimens of *Benthesicymus tanneri* were captured during sampling operations off the west coast of the Baja California Peninsula (TALUD XV, July-August 2012; TALUD XVI-B, May-June 2014), in the Gulf of California (a total of nine cruises: TALUD III, September 1991; TALUD IV, August 2000; TALUD V, December 2000; TALUD VI, March 2001; TALUD VII, June 2001; TALUD VIII, April 2005; TALUD IX, November 2005; TALUD X, February 2007), and off the SW coast of Mexico, from Jalisco to Guerrero (TALUD XII, March-April 2009). During these cruises, a total of 228 localities were sampled, from 377 to 2394 m depth. Positional coordinates for each sampling station were obtained using a GPS navigation system. Depth was measured with an EdoWestern analogic recorder (TALUD III-VIII) or a digital recorder (TALUD IX-XVI-B). All the specimens were captured with benthic gear, including an Agassiz dredge (2.5 m width, 1 m high) and a standard benthic sledge (2.35 m width, 0.9 m high), both equipped with a modified shrimp net (ca 5.5 cm stretched mesh size) with a ca 2.0 cm (3/4”) internal lining net. The material collected during this survey is deposited in the Regional Collection of Marine Invertebrates (EMU), at UNAM in Mazatlán, Mexico. The size (carapace length, CL) of all the specimens was measured to the nearest 0.1 mm and size distributions of *Benthesicymus tanneri* were explored by sex for the entire population sample in the Mexican Pacific. Sexual differences in CL were tested using a Mann-Whitney *U* test (Mann and Whitney 1947). Abbreviations are: St., sampling station; CL, carapace length; M, male; F, female; AD, Agassiz dredge; BS, benthic sledge.

## Results

### Benthesicymidae Wood-Mason, 1891

#### Benthesicymus tanneri

Taxon classificationAnimaliaDecapodaBenthesicymidae

Faxon, 1893

Figures 2 , 3 , 4 , 5 , 6

##### Material examined.

**Figure 1.**
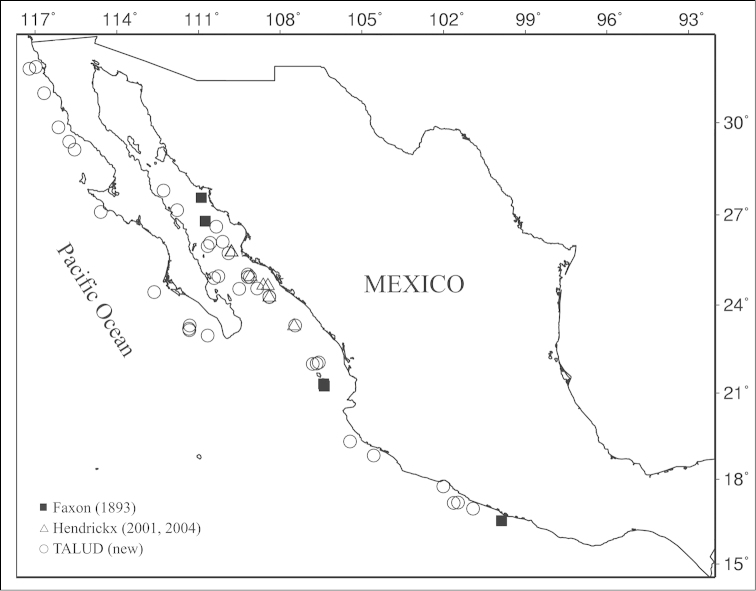
Localities in the Mexican Pacific where *Benthesicymus tanneri* Faxon, 1893 has been collected, including the TALUD project sampling stations and the localities corresponding to the type material collected during the “Albatross” cruises and used by Faxon (1893).

Specimens of *Benthesicymus tanneri* were collected in 44 of the 228 stations visited during the survey (Figure 1).

TALUD III. Material reported by Hendrickx (2001). Additional material. St. 14A (24°38'48"N; 108°26'54"W), Aug 19, 1991, 1M (CL 32.5 mm), AD, 1016–1020 (EMU-4418); St. 14B (24°39'12"N; 108°37'48"W), Aug. 19, 1991, 1F (CL 31.9 mm), AD, 1188–1208 m (EMU-2609); St. 17 (24°33'0"N; 108°50'54"W), Aug 19, 1991, 1M (CL 22.1 mm), AD, 770 m (EMU-4417); St. 24A (25°45'12"N; 109°46'48"W), Aug 24, 1991, 2M (CL 29.0–30.8 mm), AD, 1027–1060 m (EMU-100).

TALUD IV. Material reported by Hendrickx (2001).

TALUD V, St. 5 (22°0'57"N; 106°40'0"W), Dec 13, 2000, 1F (CL 36.3 mm), BS, 1515–1620 m (EMU-5540-A); St. 6 (22°N; 106°48'5"W), Dec 13, 2000, 1F (CL 41.1 mm), BS, 1950–2010 m (EMU-5540-B); St. 19 (23°17'30"N; 107°29'51"W), Dec 15, 2000, 1M (CL 31.1 mm), 3F (CL 29.1–36 mm), BS, 1180–1200 m (EMU-5523-A); St. 26 (24°15'18"N; 108°24'6"W), Dec 16, 2000, 2M (CL 29–30.7 mm), 2F (CL 32–34.2 mm), BS, 1280–1310 m (EMU-5523-B).

TALUD VI, St. 12 (23°18'36"N; 107°26'56"W), Mar 14, 2001, 1M (CL 32.5 mm), 1F (CL 34.8 mm), BS, 1050–1160 m (EMU-5539-A); St. 19 (24°16'24"N; 108°24'18"W), Mar 15, 2001, 1F (CL 50.4 mm), BS, 1160–1200 m (EMU-5539-B); St. 26 (24°56'18"N; 109°6'42"W), Mar 16, 2001, 1M (CL 33.4 mm), 1F (CL 25.2 mm), BS, 1190–1270 m (EMU-5997-A); St. 27 (25°1'12"N; 109°11'36"W), Mar 16, 2001, 1F (CL 32.3 mm), BS, 1580–1600 m (EMU-5539-C); St. 34 (25°43'50"N; 109°53'59"W), Mar 17, 2001, 1M (CL 31.9 mm), 2F (CL 3025–33.6 mm), BS, 1240–1270 m (EMU-5997-B), and 7M (CL 31.4–34.8 mm), 12F (CL 30.5–42.5 mm), and 3 unsexed specimens (14.5–21.4 mm).

TALUD VII, St. 4 (22°3'18"N; 106°34'42"W), Jun 5, 2001, 1F (CL 37.8 mm), BS, 1190 m (EMU-5541); St. 19 (24°16'12"N; 108°23'42"W), Jun 7, 2001, 1M (CL 11.2 mm) and 1F (CL 34.7 mm), BS, 1160–1180 m (EMU-6004-A); St. 33B (26°6'30"N; 110°6'42"W), Jun 9, 2001, 1F (CL 23.0 mm), BS, 1260–1300 m (EMU-6004-B).

TALUD VIII, St. 10 (24°58'12"N; 110°16'6"W), Apr 17, 2005, 1M (CL 30.4 mm), and 1F (CL 11.2 mm), BS, 1500 m (EMU-8143); St. 3 (24°32'36"N; 109°30'30"W), Apr 16, 2005, 2M (CL 31.9–34.7 mm), 3F (CL 29.2–35.7 mm), BS, 1100 m (EMU-8147).

TALUD IX, St. 20B (25°58'7"N; 110°40'4"W), Nov 14, 2005, 2F (CL 33.7–36.2 mm), BS, 1229–1343 m (EMU-8236).

TALUD X, St. 10 (27°50'5"N; 112°10'7"W), Feb 10, 2007, 1F (CL 32.3 mm), BS, 1399–1422 m (EMU-8030); St. 18 (27°9'6"N; 111°46'54"W), Feb 12, 2007, 1F (CL 31.3 mm), BS, 1526 m (EMU-8118); St. 30 (26°36'50"N; 110°21'10"W), Feb 15, 2007, 1M (CL 29.9 mm), BS, 1203–1213 m (EMU-8203).

TALUD XII, St. 5 (16°58'28"N; 100°55'20"W), Mar 28, 2008, 1F (CL 53.3 mm), BS, 1925–1977 m (EMU-8872); St. 9 (17°10'15"N; 101°37'23"W), Mar 28, 2008, 6F (CL 30.1–35.3 mm), BS, 1392–1420 m (EMU-8874); St. 10 (17°11'18"N; 101°28'30"W), Mar 29, 2008, 3F (CL 21.1–38.7 mm), BS, 1180–1299 m (EMU-10500); St. 13 (17°45'16"N; 102°0'29"W), Mar 30, 2008, 1F (CL 30 mm), BS, 1198 m (EMU-8904); St. 28 (18°50'19"N; 104°34'14"W), Apr 2, 2008, 1F (CL, 38.1 mm), BS, 1101–1106 m (EMU-10499); St. 29 (19°19'37"N; 105°26'20"W), Apr 2, 2008, 1F (CL 44.7 mm), BS, 1609–1643 m (EMU-8873).

TALUD XV, St. 1 (23°18'40"N; 111°19'37"W), Aug 4, 2012, 1F (CL 40.2 mm), BS, 750–850 m (EMU-10435); same station, 5M (CL 17.9–29.1 mm) and 7F (CL 25.3–41.1 mm), BS, 750–850 m (EMU-10434); St. 2 (23°12'2"N; 111°20'50"W), Aug 4, 2012, 4M (CL 32–33.9 mm), 5F (CL 23.2–40.6 mm) and 1Juv. (CL 12.4 mm), BS, 1118–1150 m (EMU-10436); St. 3 (23°9'N; 111°20'W), Aug 4, 2012, 1F (CL 36.4 mm), BS, 1395–1465 m (EMU-10433); St. 5C (23°16'42"N; 110°54'55"W), Aug 5, 2012, 8M (CL 20.5–35.5 mm), BS, 980–1036 m (EMU-10496-A); same station 25F (CL 20.3–40.5 mm), 1M (CL 13.4 mm), BS, 980–1036 m (EMU-10496-B); St. 5F (22°58'15"N; 110°40'17"W), Aug 5, 2012, 1F (CL 39.3 mm), BS, 1035–1108 m (EMU-10432); St. 8 (24°25'48"N; 112°38'6"W), Jul 30, 2012, 1M (CL 29.8 mm), 3F (CL 23.2–41.1 mm), BS, 1212–1235 m (EMU-10431); St. 24 (27°5'42"N; 114°35'30"W), Aug 1, 2012, 2F (CL 25–32.6 mm), BS, 772–786 m (EMU-10430).

TALUD XVI-B, St. 3 (28°42'36"N; 115°50'42"W), May 23, 2014, 2F (CL 30.1–31.0 mm), BS, 1350–1365 m (EMU-10623) St. 6 (29°08'9"N; 115°33'25"W), May 24, 2014, 10M (CL 16.4–29.9 mm) and 9F (CL 16.7–29.5 mm), BS, 1004–1102 m (EMU-10498); St. 8 (29°23'28"N; 115°45'W), May 31, 2014, 1M (CL 35.4 mm), 1F (CL 27 mm), BS, 1416–1480 m (EMU-10438); St. 16 (29°51'N; 116°9'W), May 29, 2014, 4F (CL 23.2–37.2 mm), BS, 1425–1360 m (EMU-10441); St. 23 (30°56'N; 116°40'33"W), May 27, 2014, 1M (CL 33.3 mm), 2F (CL 30.1–32.7 mm), BS, 1296–1340 m (EMU-10439); St. 26 (31°46'3"N; 116°58'12"W), May 26, 2014, 1F (CL 31.4 mm), BS, 982–989 m (EMU-10437); St. 27 (31°42'21"N; 117°13'W), May 27, 2014, BS, 1394–1397 m, 1F (CL 34.7 mm) (EMU-10440) and 1 F (CL 30.5 mm) (EMU-10497).

##### Size and sex.

**Figure 2.**
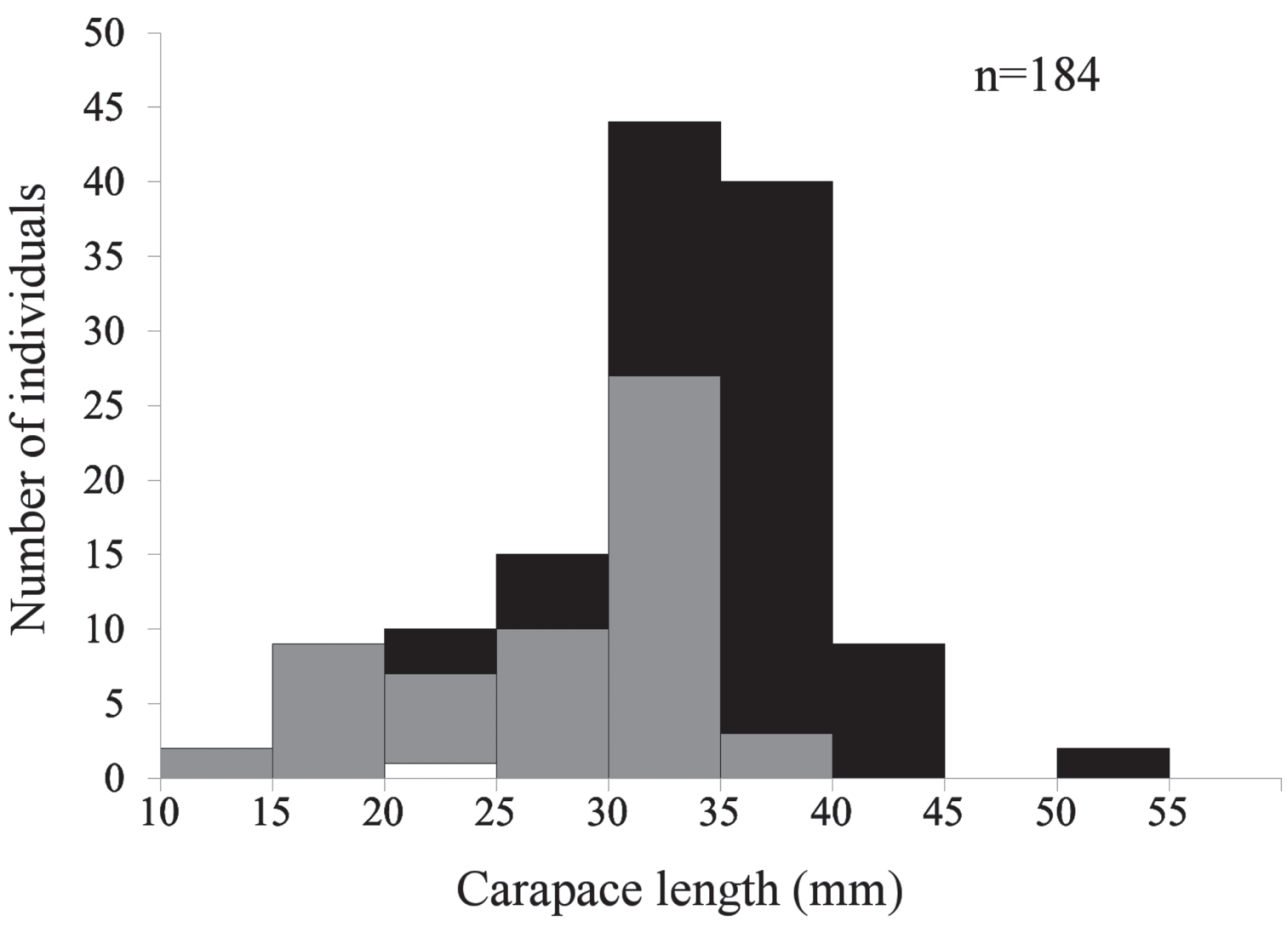
Carapace length distribution of *Benthesicymus tanneri* Faxon, 1893, by sex. White, juveniles; grey, males; black, females.

With 187 specimens available (61 males, CL 11.2–35.5 mm; 122 females, CL 16.7–53.3 mm; 3 unsexed; and 1 juvenile, CL 12.4) (M:F = 1:2), the collection of *Benthesicymus tanneri* from off western Mexico came from 44 stations and is the largest available to date for this species (Figure 1). The largest specimens measured 103 mm (male; TALUD XV, St. 5C) and 116 mm (female; TALUD XII, St. 5) total length, the latter constituting the largest specimen collected to date. The size of individuals differed across sexes (Mann-Whitney *U* test, *U*=2058.00, *p*<0.001) with females growing larger than males (Figure 2).

##### Geographic and bathymetric distributions.

The syntype series, collected by the “Albatross”, contained 56 males and 78 females (134 specimens) from 22 lots captured over a wide latitudinal range (1°3'S to 27°34'N), and included material from 4 stations in Mexico: off Acapulco and Islas Tres Marías, and in the vicinity of Guaymas (Figure 1). We are not aware of further material collected off western Mexico.

According to Wicksten (1989), Retamal and Jara (2002) and Wicksten and Hendrickx (2003), *Benthesicymus tanneri* is known from San Diego, California, USA, to Chile. The material currently examined slightly increases the distributional range of *Benthesicymus tanneri* within the Gulf of California to the north, and indicates that *Benthesicymus tanneri* occurs all along the west coast of the Baja California Peninsula where it had not been reported previously (Figure 1). In the Mexican Pacific it is a widely distributed and frequently captured species.

The material examined herein was collected between 750 and 2010 m depth with bottom sampling gear. One specimen (TALUD III, St. 17) was collected with a mid-water trawl hauled from surface to 770 m depth, in a locality where total depth was 1560 m. All species of *Benthesicymus* occur in deep water and the general depth range for *Benthesicymus tanneri* is 606–2422 m (Table 1) (Wicksten 1989).

**Table 1.** Currently known distribution, depth range and maximum size for the species of *Benthesicymus* worldwide. Species list updated according to Fransen and De Grave (2014). MW, midwater trawl; BT, benthic trawl; IK, Isaac Kid midwater trawl; AT, Agassiz (benthic) trawl.

Species	Distribution	Depth range	Size	Source
*Benthesicymus altus* Spence Bate, 1881	Eastern, central and western Pacific; Atlantic and Indian Oceans	485 m (MW); 916–4089 m; 4130 m (BT)	CL 23.5 mm; TL 120 mm	Spence Bate 1881; Wicksten 1989; Kikuchi and Nemoto 1991, Guzman and Wicksten 2000; Wicksten and Hendrickx 2003
*Benthesicymus armatus* MacGilchrist, 1905	Arabian Sea	2753 m	TL 157 mm	MacGilchrist 1905
*Benthesicymus bartletti* S. I. Smith, 1882	Atlantic, eastern Indian and western Pacific Oceans	600–5777 m	CL 34.2 mm; TL 115 mm	Crosnier 1978; D’incao 1998; Tiefenbacher 2001
*Benthesicymus brasiliensis* Spence Bate, 1881	Atlantic, southern Pacific	600–4720 m	TL 152 mm	Spence Bate 1881; Tiefenbacher 2001
*Benthesicymus cereus* Burkenroad, 1936	Atlantic	1645–1727 m	CL 25 mm; TL 76 mm	Burkenroad 1936
*Benthesicymus crenatus* Spence Bate, 1881 (type species)	Northwestern and central Pacific	3530 m (BT); 3530–6350 m; 5469–9726 m	TL 200 mm	Spence Bate 1881; Komai and Komatsu 2009; Jamieson et al. 2009
*Benthesicymus howensis* Dall, 2001	Western Pacific	1325 m	CL 24.0 mm	Dall 2001
*Benthesicymus investigatoris* Alcock & Anderson, 1899	Indo-West Pacific; SW Pacific	0–1300 (IK); 1213 (AT); 580–1690 m	CL 27.5 mm; TL 89.5 mm	Kensley 1977; Kikuchi and Nemoto 1991; Dall 2001; Retamal and Moyano 2010
*Benthesicymus iridescens* Spence Bate, 1881	Atlantic Ocean	3890–6500 m	TL 150 mm; CL 47 mm	Spence Bate 1881; Crosnier 1985; Tiefenbacher 2001
*Benthesicymus laciniatus* Rathbun, 1906	Eastern Pacific	1471–3393 m	CL 42.6 mm	Wicksten 2004
*Benthesicymus seymouri* Tirmizi, 1960	Indian Ocean	1789–3716 m	CL 40–59 mm	Crosnier 1985; Pérez-Farfante and Kensley 1997
*Benthesicymus strabus* Burkenroad, 1936	Pacific Ocean	3530 m (BT)	CL 39.5 mm	Kikuchi and Nemoto 1991
*Benthesicymus tanneri* Faxon, 1893	Eastern Pacific	606–2422 m	TL 121 mm	Wicksten 1989; Wicksten and Hendrickx 2003; Hendrickx 2004
*Benthesicymus tirmiziae* Crosnier, 1978	Indian Ocean	1920–2249 m	33 mm CL, 100 mm TL	Crosnier 1978; Peréz-Farfante and Kensley 1997
*Benthesicymus urinator* Burkenroad, 1936	Indo-Pacific	1789–3716 m; 2500–4200 m; 4120 m (BT)	CL 25.0 mm	Crosnier 1985; Kikuchi and Nemoto 1991, Dall 2001

Of the 15 recognized species of *Benthesicymus* (Table 1), currently known distributions indicate that three are widespread (*Benthesicymus altus*, *Benthesicymus bartletti*, *Benthesicymus investigatoris*), one occurs in both the Atlantic and part of the Pacific (*Benthesicymus brasiliensis*), one is distributed in the Indo-Pacific (*Benthesicymus urinator*), three are restricted to the Indian Ocean (or part of it) (*Benthesicymus armatus*, *Benthesicymus seymouri*, *Benthesicymus tirmiziae*), five occur in the Pacific Ocean (*Benthesicymus crenatus*, *Benthesicymus howensis*, *Benthesicymus strabus*, *Benthesicymus laciniatus*, *Benthesicymus tanneri*; the latter two only known from the eastern Pacific), and two are restricted to the Atlantic Ocean (*Benthesicymus iridescens*, *Benthesicymus cereus*).

##### On the presence of the hepatic spine in *Benthesicymus tanneri*.

**Figure 3.**
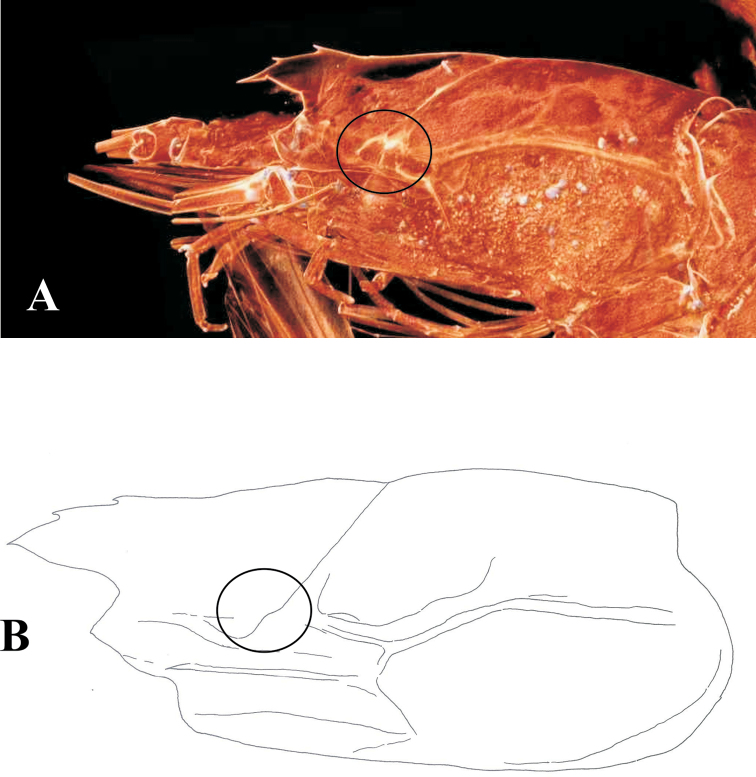
*Benthesicymus tanneri* Faxon, 1893. **A** Lateral view of syntypic specimen (MCZ-4662) **B** Lateral view of female (CL 40.6 mm) (EMU-10436). Circles indicate area where a hepatic spine is observed in some species of the genus.

In their identification key of Group II, Kikuchi and Nemoto (1991) indicated that *Benthesicymus tanneri* possesses a hepatic spine, a character that separates this species from the other four species of their Group II. Guzmán and Wicksten (2000) emphasize that the presence of a hepatic spine was not mentioned in some of the previous literature referring to *Benthesicymus tanneri* (i.e., Méndez 1981, Wicksten and Hendrickx 1992, Retamal and Soto 1993). Incidentally, the figure provided by Méndez (1981: fig. 62) does not show the presence of an hepatic spine but its reproduction in Hendrickx (1995) does (p. 437), which is an error due to the illustration process in the editorial office. In his preliminary description of *Benthesicymus tanneri*, Faxon (1893) indicated that “*Benthesicymus moratus*, Smith [S.-I. Smith, 1886, now recognized as a junior synonym of *Benthesicymus brasiliensis* Spence Bate, 1881], another allied species [of *Benthesicymus tanneri*], differs in having a distinct hepatic spine”, from which it could be concluded that the type material of *Benthesicymus tanneri* examined by Faxon (1893) lacks this spine. Re-description by Faxon (1895: 205) repeats essentially the same statement as in 1893, and his lateral illustration of the carapace (Plate H 1a) does not indicate the presence of a hepatic spine, although the lower extension of the cervical carina could easily be confused with a strong spine. Besides, this drawing does not include the presence of the pterygostomial spine either, which is definitively present in *Benthesicymus tanneri* (see Burkenroad 1936: 52). Revision by Dr. Rafael Lemaitre of part of the material used by Faxon (1893, 1895) in his syntypic series and deposited at the National Museum of Natural History, Washington, DC (USNM 21214; syntypes from the Gulf of California, Mexico) confirms the fact that there is no trace of a hepatic spine on the specimens examined. Another revision by Adam Baldinger of one of the syntypes of *Benthesicymus tanneri* (MCZ-4662) deposited at the Museum of Comparative Zoology at Harvard also clearly indicates the absence of a hepatic spine (Figure 3A). An illustration of a large specimen of *Benthesicymus tanneri* collected during this survey is also provided for comparison (Figure 3B). References to this spine in earlier literature (Kikuchi and Nemoto 1991, Hendrickx 1995, Dall 2001) are therefore in error. Consequently, the groups definition presented by Kikuchi and Nemoto (1991) have to be altered because all species of Group II as defined by these authors in their key lack the hepatic spine which is otherwise present in seven of the ten species of their Group I. Moreover, the identification key proposed by Dall (2001) should be partly modified.

##### Reproductive organs.

**Figure 4.**
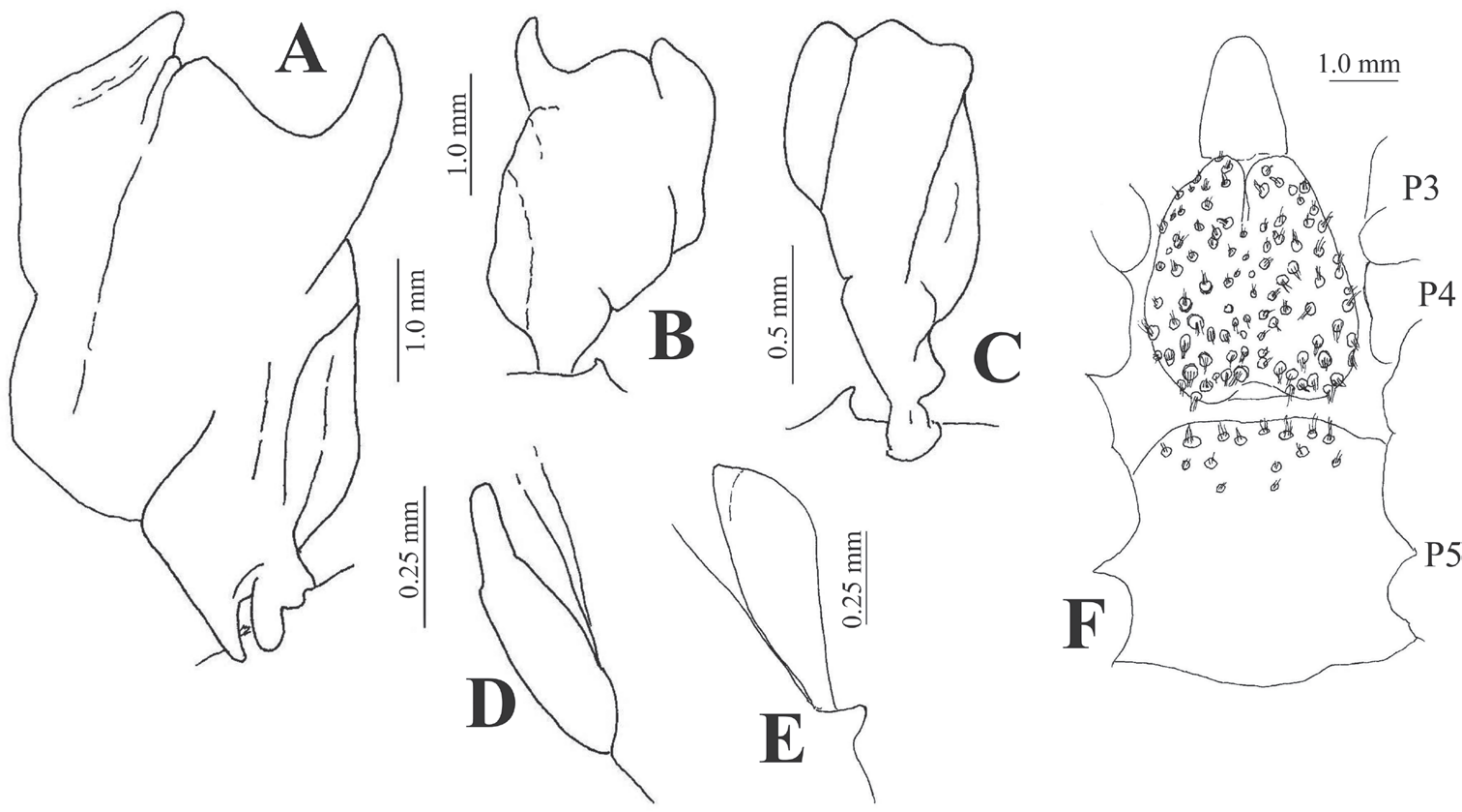
*Benthesicymus tanneri* Faxon, 1893. Anterior view of petasma (**A–E**) of males of different carapace length (**A–D** EMU-10498; **E** EMU-6004-A) and thelycum (**F**) of a mature female (EMU-10441). **A** CL 29.9 mm; **B** CL 22.3 mm; **C** CL 17.5 mm; **D** CL 16.4 mm; **E** CL 11.2 mm; **F** CL 36.6 mm.

While studying fine morphology of *Benthesicymus carinatus* (now included in *Altelatipes*), Tavares (2009) noted the lack of basic information related with the description and development of the reproductive organs of *Benthesicymus* s.l. The male petasma of *Benthesicymus tanneri* was illustrated by Faxon (1895) and by Hendrickx and Estrada-Navarrete (1996). Material examined collected in station 6 of the TALUD XVI-B cruise includes small and medium-size males with immature petasma (Figure 4A–D). The smallest male with visible petasma was 11.2 mm CL, in which a small bud without any elaborated structure could be seen. A slightly larger male (CL 16.4 mm) had a similar petasma (Figure 4D). However, another young male from station 19 of TALUD VII cruise with CL 11.2 mm (i.e., smaller than the male of Figure 4D) presented a relatively larger petasma (Figure 4E). The crescent-shape lateral process, which is typical of *Benthesicymus tanneri*, is not yet developed in males of CL 17.5 mm (Figure 4C). In a male of CL 29.9 mm the two sections (left and right) of the petasma are well developed (Figure 4B) but not yet united medially.

**Figure 5.**
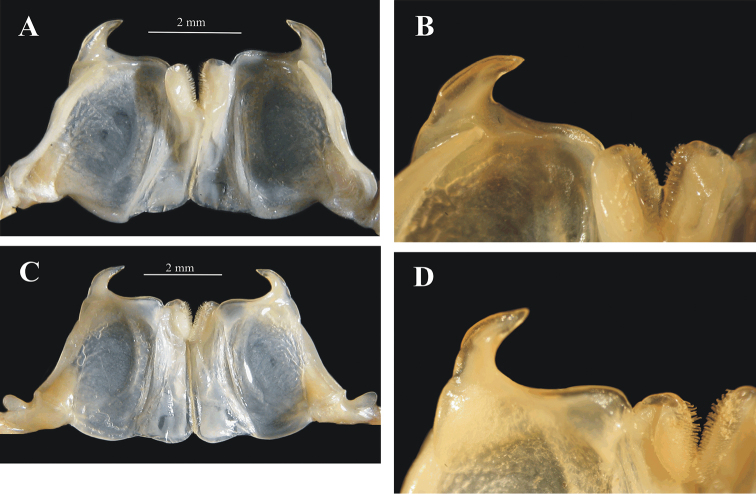
*Benthesicymus tanneri* Faxon, 1893. Petasma of a fully mature male (CL 35.7 mm) (EMU-8147) **A** Posterior view **B** Same, detail of ventral margin **C** Anterior view **D** Same, detail of ventral margin.

The fully developed petasma (Figure 5A–D) of *Benthesicymus tanneri* (CL ≥ 35 mm) is clearly distinct from known petasma of mature males of nine species of the genus in the presence of the lateral crescent-shape process. In *Benthesicymus altus*, *Benthesicymus brasiliensis*, *Benthesicymus crenatus* (the type species of the genus), *Benthesicymus investigatoris* Alcok & Anderson, 1899, *Benthesicymus iridescens* Spence Bate, 1881, *Benthesicymus laciniatus*, *Benthesicymus seymouri* Tirmizi, 1960, *Benthesicymus strabus* Burkenroad, 1936, and *Benthesicymus urinator* Burkenroad, 1936, the petasma lacks the lateral crescent-shape process (see A. Milne Edwards and Bouvier 1909, Burkenroad 1936, Crosnier 1978, 1985, Hendrickx 1996, Kikuchi and Nemoto 1991) (see below for the case of *Benthesicymus bartletti* S.I. Smith, 1882). It should be noted that figure 1, page 28, of Burkenroad (1936) is labeled “*Benthesicymus laciniatus* Rathbun”, which is most certainly an error, and this illustration likely belongs to *Benthesicymus crenatus*, as indicated earlier in the text by the author. Burkenroad (1936: fig. 35) also provided an illustration of the petasma of *Benthesicymus cereus* Burkenroad, 1936, probably a juvenile. This figure lacks a lateral crescent-shape process but, as in the case of *Benthesicymus tanneri* (see Figures 3, 4), this process may appear later during the growth of the species. Of the remaining three species of *Benthesicymus*, a crescent-like process has been described only in *Benthesicymus tirmiziae* Crosnier, 1978 (but see below). The petasma of *Benthesicymus howensis* Dall, 2001, remains undescribed as the species (originally described as a new subspecies of *Benthesicymus urinator*) is known only from the two females of the type material. We were not able to locate an illustration of the petasma of *Benthesicymus armatus* MacGilchrist, 1905. Another question remains open as far as illustrations of petasma in literature are concerned. Peréz-Farfante and Kensley (1997: fig. 27) provided an illustration of both the petasma and the thelycum of a species which certainly belongs to *Benthesicymus*; however, the figure caption is the same as the one inserted in figure 25 of the same monograph (i.e. for *Bentheogennema intermedia* (Spence Bate, 1888)) and it was therefore difficult to assess to which species of the genus this figure actually belongs to. A search by Rose Gulledge, Museum specialist at the US National History Museum, Smithsonian Institution crustacean department, Maryland, USA, was successful in finding the original plates prepared by the illustrator of Peréz-Farfante and Kensley (1997). Pencil markings and notes on the plates indicate that the petasma and thelycum of figure 27 belong to *Benthesicymus bartletti*, and that “species in book is wrong [...] must say *Benthesicymus bartletti*”. Consequently, *Benthesicymus bartletti* represents a third species featuring a crescent-shaped lateral process on the petasma, as *Benthesicymus tanneri* and *Benthesicymus tirmiziae* do.

The female thelycum of *Benthesicymus tanneri* was roughly illustrated by Faxon (1895, plate H-1b) and is illustrated herein (Figure 4F). A small tuft of setae is clearly observed arising from each minute pit of the thelycum middle plate (sternite XIII). Of the two groups of species considered by Burkenroad (1936) in his synopsis of *Benthesicymus*, Group I possesses a “thelycum without well-defined receptacles between the twelfth and the thirteenth sternites, the scutes of the twelfth and thirteenth sternites being simple and unexpanded”. Group II posseses “well-defined cavities between the twelfth and the thirteenth sternites, the scutes of the thirteenth sternites being broadly expanded to overlap the sternal surface proper”. Based on these criteria *Benthesicymus tanneri* belongs to Group II, with the scutes of sternite XIII broadly expanded (Figure 4F).

##### Color.

**Figure 6.**
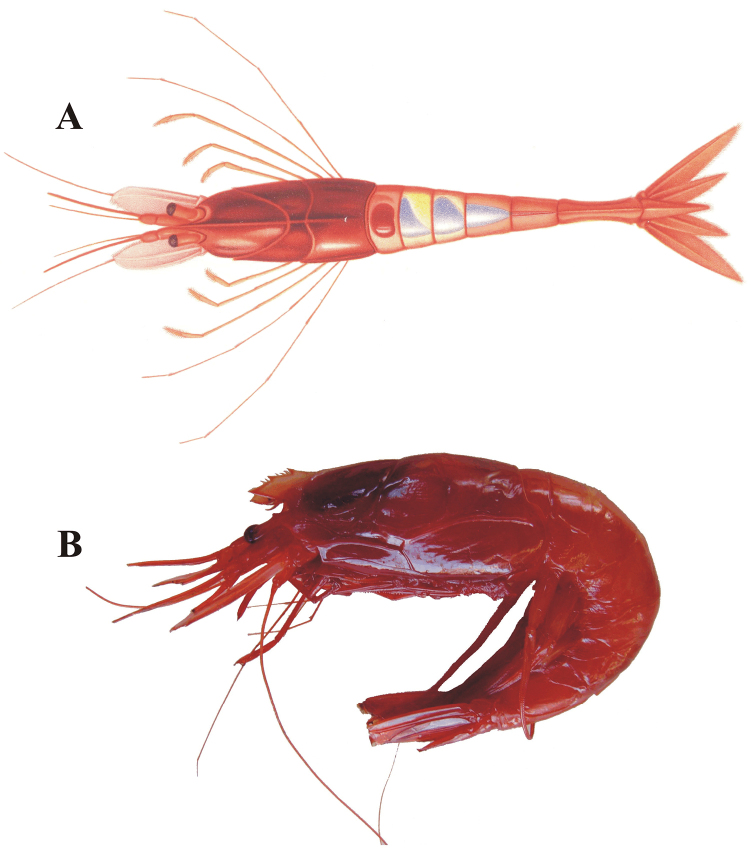
*Benthesicymus tanneri* Faxon, 1893. **A** Dorsal view of one of the syntypes used by Faxon (1893) (from Faxon 1895) **B** Fresh specimen female, CL 30 mm, lateral view (EMU-8904).

The color of fresh specimens was described by Faxon (1895: 207) and a color drawing (Plate H-1) was added to his contribution (reproduced here as Figure 6A). All specimens collected during the TALUD survey presented the typical “deep red” color (Figure 6B) described by Faxon (1895). The large patch of bright blue color on the back of the abdominal somites 2–4 mentioned by Faxon (op. cit.) and also observed by Moscoso (2012) actually corresponds to the gonads of mature specimens that extend backward from the thoracic area (pers. observ.).

##### Fishery resource.

Although it reaches a size (i.e., over 115 mm total length) comparable with other species of Dendrobranchiata used as food, *Benthesicymus tanneri* is not currently subject to any commercial exploitation. It has been considered a potential fisheries resource for the area (see Hendrickx 1995) to a large extent because it occurs together with other species of established potential for deep-water fisheries (e.g., *Heterocarpus affinis* Faxon, 1893, *Haliporoides diomedeae* Faxon, 1893) (Barriga et al. 2009). Since 2004, the Peru fishery program has included *Benthesicymus tanneri* in a short list of sub-exploited deep-water shrimps subject to “exploratory fishing” in Peruvian waters (Ministerio de la Producción 2004). In the specific case of the western central Pacific, Chan (1998) reported the presence of six species of *Benthesicymus* in this area, but none was considered of importance to fishery, even as a potential resource, probably because this genus has nowhere been reported to be abundant. The 15 species of *Benthesicymus* known to date are from mid-sized (from ca 70–80 mm TL) to large (ca 200 mm TL) (Table 1) but are all from deep-water, thus rending any exploitation attempt very complex.

### Key to the species of *Benthesicymus* from the eastern Pacific

**Table:** 

1a	Posterolateral margin of fifth abdominal somite with small spines	***Benthesicymus laciniatus***
1b	Posterolateral margin of fifth abdominal somite without spines	**2**
2a	Petasma ventral margin strongly convex, without lateral crescent-shape process. Thelycum sternite XIII plate smooth, without small pits and setae	***Benthesicymus investigatoris***
2b	Petasma ventral margin straight to slightly concave, with or without lateral crescent-shape process. Thelycum sternite XIII plate bearing small pits	**3**
3a	Petasma with strong ventrolateral crescent-shape process. Thelycum sternite XIII plate longer than wide, shallow anterior notch	***Benthesicymus tanneri***
3b	Petasma without ventrolateral crescent-shape process. Thelycum sternite XIII plate wider than long, deep anterior notch	***Benthesicymus altus***

## Supplementary Material

XML Treatment for Benthesicymus tanneri
